# The Phytologist, a Popular Botanical Miscellany

**Published:** 1847-10

**Authors:** 


### Art. XIII.

1. *The Phytologist, a Popular Botanical Miscellany.* Conducted by George Luxford, a.l.s., f.b.s.e.—*London,* 1841-7.

2. *The Zoologist, a Popular Miscellany of Natural History.* Conducted by Edward Newman, f.l.s., z.s., &c.—*London,* 1843-7.

The works above mentioned are issued as monthly journals, and are, for the objects for which they are intended, works of very considerable merit; and though not bearing on the practice of our profession, and so far not constituting that class of work, which as a general rule, we consider the more proper objects of notice in these pages, yet as treating on subjects which are or ought to be interesting to all the members of our profession, we are induced to give them a passing notice, and the more so as we believe they have not received from medical men in general, that attention and support to which we think they are entitled from a class of men whose education and tastes naturally bear on the subjects of which they treat. Though treating on distinct branches of natural history, yet, as being conducted in the same spirit, and being sister productions, and owing their origin to the same spirited individual, who is the proprietor of both as well as the editor of the 'Zoologist,' we feel, on these accounts, disposed to consider them together. Their object is to afford to the numerous class of those who are interested in the subjects of zoology and botany, an opportunity of perusing, at small cost, a readable account of the observations and findings of the day, and of recording their own observations on the same subjects. The pages of these works are open to the small facts which must of necessity be excluded from the elaborate transactions of societies, but which possess an interest for many, who, with the true love of the contemplation and observation of nature, have neither time nor inclination for abstruse researches. To the active members of our profession, who in the course of their studies have acquired a taste for these subjects, but who would find a sustained attentive study of them incompatible with the important and anxious duties of active practice, the works before us are peculiarly adapted,—recording facts interesting to all, and offering their ready pages to observers who may be willing to record what passes before their eyes, but who would shrink from the task of an elaborate paper.

We must be careful, however, by the above remarks, not to mislead by giving the idea that we have only before us the results of the "*'prentice hands*" of natural history tyros. Open, as are these works, to *all*, yet in the table of contents amongst the names of the *unknown*, we see also those of our well-known and justly celebrated authors, who here record the facts, which, though valuable in themselves, could not find admittance to the manual or condensed synopsis. *Facts,* however, are the words of nature, and whether recorded by the unknown Mr. A. or the celebrated Professor B. if faithfully related, speak the same lesson, and possess an equal value: and in accordance with this view, it is gratifying to find how small a portion of nonsense finds its way to the open pages of these liberal journals, the preface of one of which declares (Zool. vol. i, p. 6,) that "every one who describes a single fact is welcome,—nay more than that,—has a claim to be admitted as a contributor."

*The Phytologist* we shall notice first as being the elder sister, though we believe hitherto the less sought. Its immediate origin and object we quote from the proprietor's preface.

"The Phytologist owes its existence to the desire of recording and preserving FACTS, OBSERVATIONS, and OPINIONS relating to botany in general, but more especially to British botany. Prior to its commencement *these* had no appropriate receptacle. There was no periodical to which they would be acceptable. For works of a general character they were esteemed too dull; for those of high scientific pretensions they were supposed too trifling. By field-botanists alone were they considered worth preserving: to such the utility, the value of an unpretending monthly journal was most manifest; and these field-botanists—these observers—these labourers in the delightful field of botanical inquiry, have freely availed themselves of its pages: they have done all that was anticipated, and 'the Phytologist' has become the medium of their communications with each other and with the botanical public." (vol. i, p. v.)

The above we have extracted from the preface of the first volume; in that of the last we are informed that its aim is that it should be "a complete register of the botany of Britain," and that with this object information given to other journals is invariably *noticed* in the 'Phytologist.' From an intimate acquaintance with the work from its commencement, we may say that it has from the first fulfilled the intention thus only recently expressed. All newly-discovered species of plants during the last few years, may be found here recorded; and considering how many years British botany has been cultivated, and since the earlier days of Smith, with only an occasional addition to our flora, the number which have of late years been added is truly surprising, and marks the habit of careful analysis, which characterizes the present generation. In addition to these matters of detail, interesting, most interesting, to the collecting botanist, are numerous very readable excursions, and some truly scientific papers on the philosophy of botany, the latter chiefly from the pen of Mr. Hewett Watson, the well-known author of the 'Geography of British Plants;" while with regard to the material for the further consideration of the interesting subjects of the last-named work, ample funds may be here found from all parts of Britain.

A very remarkable fact is recorded by Mr. Spence, (vol. ii, p. 85,) of small branches growing from the disk of a leaf in *Jungermannia juniperina,* we extract the account from the observer's own words.

"I noticed that, at the summit of one of the stems, the leaves were so closely crowded together as to form a sort of coma; and that, from amongst these leaves, which were spread out on every side and recurved, there proceeded several minute branchlets, some nearly erect, others gracefully pendulous, resembling in miniature the flagelliform ramuli of Mnium undulatum, *Hedw.* I immediately placed the specimen under the microscope, and proceeded to dissect it; but what was my surprise to find that the branchlets issued from the coma, instead of being attached to the main stem, *actually grew on the surface of the leaves*, where they had all the appearance of having been stuck by art!........They were clad with leaves and stipules, precisely as those of the stem.........The base of each branchlet is slightly dilated into a sort of bulb, which is affixed to the leaf by a very small surface (covering exactly one cellule), and when detached by gentle force, it brings away with it the upper paries of the cellule." (Phytol. ii, p. 85.)

The case is interesting as illustrating that, independently of buds, seeds, and spores, mere nucleated vesicles may in certain cases reproduce all the vegetable organs, a fact now becoming extensively made use of by gardeners who are succeeding in propagating many plants by mere leaves or part of leaves.

We have some interesting remarks (vol. ii, pp. 87, 116) on the subject of the Œnanthe crocata, of which so many instances are on record of its destructive poisonous results in England, while Dr. Christison affirms that in Scotland the plant is not poisonous at all. The existence of the yellow juice from which the species is named is equally capricious. From the limited observations here recorded, it would appear that the yellow juice and active poisonous properties are coincident.

There are numerous papers on monstrosities, illustrative of the theory of the shrewd and philosophical Göthe, on the metamorphoses of the parts of plants or *morphology*, as it is now termed, and also some very elaborate articles on vegetable embryology (vol. i, pp. 625, 675, 731, 881; vol. ii, pp. 914-22.)

The subject of the potato disease has given rise to many communications, some of which (vol. ii, pp. 528, 631, 880) are of considerable interest, and in particular, one of the best we have anywhere seen on this subject, is a communication by Dr. Tyerman, of Bodmin, read before the Royal Cornwall Polytechnic Society, and published in this Journal (vol. ii, p. 826). We fear, however, that the phytologists' communications, like those which have appeared elsewhere, still leave this very mysterious subject in a most unsatisfactory position. The supposition of Mr. Smee, of the influence of an aphis in producing this result, scarcely needs a serious denial; and, on the other hand, though the universal existence of a vegetable parasite (*Botrytis infestans*) be observed, and though it be conceded that it is a true parasite, inasmuch as it subsists on living and not dead vegetable matter, yet for its finding a suitable pabulum, we are told the plant must be in a feeble condition, and it is this *aliquid prius* which, in our opinion, is *the* disease, while, to use the words of Mr. Ward (vol. ii, p. 816), the fungi are, "in fact, the result and not the cause." The explanation of this catastrophe on the untenable and unphilosophical theory of Knight, that plants propagated by division all die off together, is now happily receiving (vol. ii, p. 911) the best refutation in the great diminution, if not the total extinction of the disease this year. This disease has its parallel in the epidemics of man and animals. It has no *affinity* with these, but they are nevertheless its true *analogues*.

During the last two years an arrangement has been made by the Botanical Society of London with this Journal for the publication in its pages of the Papers read before the Society, which are appointed by the council for publication in the Transactions, by which they both enrich this work and receive a wider circulation.

We feel tempted to enter into the popular subject of progressive development, as relating to botanical subjects, on the limits of species, or on the debated value of certain well known forms of plants whether as being real species or only varieties, subjects on which we have ample matter in the work before us, but to dwell on which we feel would be foreign to our purpose.

We cannot, however, take leave of the 'Phytologist' without noticing the excellence of the wood engravings with which many of the communications are executed, and especially some very excellent articles on our Ferns from the pen of the proprietor of the work who is peculiarly strong on this point; his beautiful work on 'British Ferns' being our best monograph on the subject. We now pass on to make a few remarks on the other work.

*The Zoologist.* This work, though more recently commenced, has already proved to be the more popular. The illustrations on wood are beautifully executed and very numerous. The subject which more than any other appears to have received the most copious contributions is the very interesting one of the migration of birds, on which the pages of the Zoologist contain a mass of matter affording considerable materials from which to arrive at the knowledge of this very curious fact.

The plumage of birds, as varying by age, has received some attention from some of the contributors, and the contributions noticing the occurrence of various of the rarer insects offer very materially towards furnishing *data* for a very improved knowledge of their geographical distribution.

In a recent communication (p. 1637) we find it stated, that it is the parent cuckoo who ejects from the nest the offspring of the rightful owner, and not the young cuckoo himself. "This," we agree, "differs widely from the common opinion and deserves closer investigation."

Mr. Newman, the editor, has just added some valuable observations in confirmation of a most interesting communication read by Mr. Newport, before the Linnæan Society, in November 1845 (reported p. 1241). It appears by the observation of these gentlemen that certain *coleoptera,* prior to their existence in the ordinary form of the larvæ in this order, first emerge from the ova, or are produced viviparously as active hexapods, which immediately become parasitic on other insects, by which means they are conveyed to the localities—the nests of various bees, for instance—where their proper nourishment is provided, and where, assuming the form of nearly apodous maggot-like larvæ, they acquire their full dimensions, and after the usual transformations emerge as perfect insects. This is a very curious fact, and is particularly striking when viewed in relation to the important details lately brought before the world in that most interesting work by Steenstrup, 'On the Alternation of Generations,' recently issued by the Ray Society.* It appears, moreover, that one link in the chain of observations, as recorded by Messrs. Newport and Newman, is yet wanting, and that the mode of transition from the active parasite to the lazy maggot-larva has not yet been observed. We confess we

* On the Alternation of Generations; or, the Propagation and Development of Animals through Alternate Generations: a peculiar mode of fostering the young in the lower classes of animals. By Joh. Japetus Sm. Steenstrup, Lecturer in the Academy of Söro. Translated from the German version of C. H. Lorenzen, by George Busk.—London, printed for the Ray Society, 1845.

should not be surprised if this should prove an instance of true alternation, and not a *mere* metamorphosis, and that the little parasite is the nurse (*amme*) of the subsequent larvæ.

We find (vol. i, p. 175) several instances recorded of the fact of insects of different species being found *in copula:* this occurrence between *Smerinthus Populi* and *Sm. ocellatus* is mentioned by two observers, and by one of these the hybrid results were obtained. In the same page is recorded the yet more remarkable fact of insects of different *genera* being so found, and these, though of the same tribe, yet insects of such different conformation and habits as *Sphinx Ligustri* and *Smerinthus ocellatus,* "and what renders this the more singular is, that there were several individuals of both sexes of the two species at the same time in the cage." This fact is of importance, as showing that the circumstance of insects being found *in copula* ceases to be of any value for the purpose of deciding, with variable insects, whether certain forms be of the value of species or varieties only. While speaking of insects, we may notice another fact here recorded, (vol. iii, p. 1198,) which is also very interesting; it has reference to the effect of light and colour on caterpillars. *Vanessa Io* was the species chosen for experiment, and numerous individuals were placed in four compartments of a box, one darkened, another having common light, and the others lighted, one through blue and the other through yellow glass. Those in the yellow light appeared less healthy but all formed their chrysalis at the same time; they emerged, however, thus:—"those in the dark all made their appearance first (August 12 and 13), and were all out before a specimen had appeared in the other three cells. Next came those in the common light (August 14 and 15); then the blue and yellow together (August 15—20).

The anatomy of the horse-leech is very elaborately described by the well-known microscopist, the late lamented Mr. John Queckett. As the following passages are interesting we transcribe them:

"The jaws themselves are firmly imbedded in strong muscles which are disposed in such a manner as to move them backwards, and forwards as in the act of biting, with great force and rapidity. . . . . . . But the dental apparatus of the medicinal leech is much more marked than in this species, and this fact, combined with the striking differences in their alimentary canals (the medicinal leech being furnished with very large *cæca*, which in this are little more than rudimentary,) affords a most satisfactory explanation of the reason why the horse-leech is unfit for medicinal purposes, for instead of the fifteen blunt molar-like teeth, with which the latter is provided, the jaws of the medicinal species are much larger, more convex, and of very much firmer consistence. Each jaw is provided with a row of teeth, varying from sixty to eighty in number; their points or apices are much sharper than those of the finest needles; they are likewise strongly imbedded in the cartilaginous matter of the jaw; in the horse-leech, on the contrary, the teeth appear to be *loosely connected* to the upper part of the jaw." (Vol. i, p. 21.)

In conclusion, we unhesitatingly recommend these works to our readers, and we consider that all who are interested in their respective subjects are very much indebted to Mr. Newman's spirited conduct in originating them, and in persevering till they are become established on a firm footing, as well as for offering them at a price so low as not to be a consideration with any.

